# Accelerated pre‐senile systemic amyloidosis in PACAP knockout mice – a protective role of PACAP in age‐related degenerative processes

**DOI:** 10.1002/path.5100

**Published:** 2018-07-04

**Authors:** Dora Reglodi, Adel Jungling, Rémi Longuespée, Joerg Kriegsmann, Rita Casadonte, Mark Kriegsmann, Tamas Juhasz, Sebastian Bardosi, Andrea Tamas, Balazs Daniel Fulop, Krisztina Kovacs, Zsuzsanna Nagy, Jason Sparks, Attila Miseta, Gabriel Mazzucchelli, Hitoshi Hashimoto, Attila Bardosi

**Affiliations:** ^1^ Department of Anatomy, MTA‐PTE PACAP Research Group University of Pecs Medical School Pécs Hungary; ^2^ Institute of Pathology University of Heidelberg Heidelberg Germany; ^3^ Center for Histology Cytology and Molecular Diagnostics Trier Germany; ^4^ Proteopath GmbH Trier Germany; ^5^ Department of Anatomy, Histology and Embryology, Faculty of Medicine University of Debrecen Hungary; ^6^ Department of Biochemistry and Medical Chemistry University of Pecs Medical School Pécs Hungary; ^7^ Second Department of Internal Medicine University of Pecs Medical School Pécs Hungary; ^8^ Department of Laboratory Medicine and Szentagothai Research Centre University of Pecs Medical School Pécs Hungary; ^9^ Laboratory of Mass Spectrometry (LSM) – MolSys, Department of Chemistry University of Liège Belgium; ^10^ Laboratory of Molecular Neuropharmacology, Graduate School of Pharmaceutical Sciences Osaka University Japan

**Keywords:** amyloid, apolipoprotein‐AIV, MALDI imaging, proteomic analysis

## Abstract

Dysregulation of neuropeptides may play an important role in aging‐induced impairments. Among them, pituitary adenylate cyclase‐activating polypeptide (PACAP) is a potent cytoprotective peptide that provides an endogenous control against a variety of tissue‐damaging stimuli. We hypothesized that the progressive decline of PACAP throughout life and the well‐known general cytoprotective effects of PACAP lead to age‐related pathophysiological changes in PACAP deficiency, supported by the increased vulnerability to various stressors of animals partially or totally lacking PACAP. Using young and aging CD1 PACAP knockout (KO) and wild type (WT) mice, we demonstrated pre‐senile amyloidosis in young PACAP KO animals and showed that senile amyloidosis appeared accelerated, more generalized, more severe, and affected more individuals. Histopathology showed age‐related systemic amyloidosis with mainly kidney, spleen, liver, skin, thyroid, intestinal, tracheal, and esophageal involvement. Mass spectrometry‐based proteomic analysis, reconfirmed with immunohistochemistry, revealed that apolipoprotein‐AIV was the main amyloid protein in the deposits together with several accompanying proteins. Although the local amyloidogenic protein expression was disturbed in KO animals, no difference was found in laboratory lipid parameters, suggesting a complex pathway leading to increased age‐related degeneration with amyloid deposits in the absence of PACAP. In spite of no marked inflammatory histological changes or blood test parameters, we detected a disturbed cytokine profile that possibly creates a pro‐inflammatory milieu favoring amyloid deposition. In summary, here we describe accelerated systemic senile amyloidosis in PACAP gene‐deficient mice, which might indicate an early aging phenomenon in this mouse strain. Thus, PACAP KO mice could serve as a model of accelerated aging with human relevance. © 2018 The Authors. *The Journal of Pathology* published by John Wiley & Sons Ltd on behalf of Pathological Society of Great Britain and Ireland.

## Introduction

Previous findings indicate that PACAP progressively declines during aging and its deficiency substantially increases tissue vulnerability 1, 2. Here, we hypothesize that PACAP deficiency leads to age‐related systemic degeneration, causing accelerated pre‐senile systemic amyloidosis, as a representation of the aging‐related chronic condition (physiological as well as disease‐related) in mice.

Amyloidosis is a heterogeneous group of diseases, from small local to generalized systemic forms, characterized by the deposition of amyloid fibrils caused by inappropriate protein aggregation 3, 4, 5, 6, 7. Many forms affect the brain (such as Alzheimer's and Parkinson's disease), while others leave the CNS intact 3, 8. Advances in proteomics have revealed more than 30 different proteins prone to fibril formation in the complex amyloid matrix 6, 9, 10, 11, 12, 13. Excessive production, mutations or proteolytic digestion can result in an amyloidogenic protein form, the fibril formation of which requires several factors, such as proteases, nucleating particles, chaperons, matrix molecules, and microenvironment 10, 13, the balance of which can be disturbed in aging, when senile amyloidosis occurs in several mouse strains as well as in humans 8.

Natural peptides or peptide‐based molecules preventing amyloid formation or its progression have been recognized 3, 14 as molecules with possible interference in amyloidogenic mechanisms including stabilizing non‐toxic amyloid species, redirecting aggregation pathways, trapping toxic amyloid species or retaining native protein conformation 15. In turn, dysregulation of neuropeptides may play a role in aging‐induced impairments and accelerate the formation of amyloid deposits 16. PACAP is a highly effective neuro‐ and general‐cytoprotective peptide that provides endogenous control in tissue damage by exerting a unique combination of simultaneous promotion of anti‐inflammatory, anti‐apoptotic, and antioxidant pathways mainly via PAC1 receptor and VPAC1/2 receptors 1, 2, 17, 18, 19, 20, 21.

PACAP decline has been reported in aging human and monkey brains 22, 23 and in aging rat cerebrovascular cells, where decreased release of endothelial PACAP upon oxidative stress can be observed 24, 25. Lack of endogenous PACAP results in a variety of abnormalities 2, 18; for example, a lower level or absence of PACAP leads to increased vulnerability and accelerated aging, as shown in the retina and by the reduced antioxidant capacity of aging KO mice 22, 26, 27. These alterations may lead to the reduced life span and increased mortality (approximately twice as high as in WT mice) of mice lacking PACAP, as observed in our PACAP KO colony. In PACAP KO mice, mass spectrometric (MS) analysis uncovered an altered homeostatic protein pattern resulting in disturbed energy balance, the compensation of which is compromised under a challenged environment and possibly during aging 28. Our main hypothesis is that PACAP deficiency mimics premature aging with pre‐senile systemic degenerative changes. Here, we report the results of detailed systemic histopathological analysis showing accelerated systemic amyloidosis in mice lacking PACAP. MS‐based proteomic analysis of deposits identified apolipoprotein (apo)‐AIV‐type (Apo‐AIV) amyloidosis with several other amyloidogenic components. All amyloid‐associated proteins were further investigated by matrix‐assisted laser desorption/ionization (MALDI) imaging MS (IMS) to directly map the distribution of corresponding peptides in tissue.

## Materials and methods

### Animals

The generation of KO mice on a CD1 background has been described previously 29. Maintenance, backcrossing for ten generations of the in‐house‐bred KO mice, and all procedures were performed with permission (BA02/2000‐24/2011; BA02/2000‐20/2006 29, 30). WT (*n* = 29) and homozygous PACAP KO mice (*n* = 29) were grouped into 3‐ to 12‐ (young) and 13‐ to 24‐month‐old (aging) groups. Genotyping from tail samples was performed at sacrifice using a Phire Animal Tissue Direct PCR Kit (Thermo Fisher Scientific, Waltham, MA, USA) 30.

### Histological analysis

Mice (*n* = 15 WT, 15 KO) were killed under isoflurane anesthesia (AErrane, Baxter, Budapest, Hungary) and removed organs (see supplementary material, Supplementary materials and methods for a detailed list) were placed in 4% buffered paraformaldehyde and embedded in paraffin wax, and 3‐μm‐thick sections were cut and stained with hematoxylin and eosin (Paraform Sectionable Cassette System, Tissue‐Tek X‐Press, AutoTek120 and Prisma Film Coverslipper HQ, smart automation system – Sakura Finetek, Alphen aan den Rijn, The Netherlands). Parallel sections were stained with Congo red, the gold standard for amyloid fibrils 31, which displayed amyloid deposits in red‐orange under normal light, whereas apple‐green birefringence was indicative of amyloid under polarized light. A semi‐quantitative scoring of Congo red‐positive deposits from 0 to 3 was performed according to pathological criteria 32, 33: amyloid index 0: no amyloid; 1: slight focal; 2: moderate/severe focal or slight diffuse; 3: massive diffuse amyloid deposit. As no gender differences were observed, scores are presented combined 32. Percentages of affected individuals for each organ were also evaluated. Statistical comparison between WT and KO mice was made with a non‐parametric Mann–Whitney test. For comparison between age groups, the Kruskal–Wallis test and Dunn's multiple comparison test were used.

### Laser microdissection (LMD)‐based microproteomic analysis

Sections of intestinal villi with massive amyloidosis were chosen for LMD‐based microproteomic analysis to characterize the type of amyloidosis. Characterization was performed using a recently developed method for proteomic analysis of small laser‐microdissected formalin‐fixed, paraffin‐embedded (FFPE) samples 34, 35, 36. The procedure for LMD‐based microproteomics has been described previously 37 and uses in‐solution tryptic digestion of proteins directly from tissue pieces. The released peptides are then separated by reverse‐phase liquid chromatography directly bridged to a quadrupole‐Orbitrap mass spectrometer (see supplementary material, Supplementary materials and methods for details). MaxQuant version 1.5.2.8 was used for raw file analysis.

### MALDI IMS

Intestine was selected as it contained abundant deposits in well‐circumscribed areas in the villi. Five‐micrometer‐thick sections were cut from two different FFPE specimens and mounted onto conductive indium tin oxide (ITO)‐coated glass slides (Bruker Daltonik, Bremen, Germany).

Sample processing consisted of deparaffinization of the tissue sections, antigen retrieval, on‐tissue spraying of trypsin and tissue digestion, and finally matrix deposition (see supplementary material, Supplementary materials and methods for details).

MS measurements were carried out using a rapifleX MALDI Tissuetyper (Bruker Daltonik, Bremen, Germany) instrument at 50 μm spatial resolution from *m/z* 640 to *m/z* 3000. External calibration was performed using Peptide calibration standard II (Bruker Daltonik). FlexImaging 5.0 (Bruker Daltonik) was used to visualize ion images; all displayed intensities were normalized to total ion count (TIC). Further data analysis was performed with SCiLS Lab 2018b software (SCiLS, Bremen, Germany).

### Immunohistochemistry for Apo‐AIV and molecular analysis

We performed immunostaining using an anti‐Apo‐AIV antibody (1:200, catalog number NBP1‐06019; Novus Biologicals, Littleton, CO, USA) to confirm the presence of Apo‐AIV, the main amyloid component, in tissues with abundant deposits: intestine, kidney, liver, and spleen. Staining was performed using a Ventana BenchMark XT Ultra (Ventana Medical Systems, Inc, Tucson, AZ, USA). A method control was performed by omitting the primary antiserum, and which produced no staining. PCR for amyloid protein mRNAs was performed from intestinal samples from aging WT and KO mice (*n* = 3 per group) with confirmed amyloidosis. In addition, kidney homogenates (*n* = 4 per group) were processed for cytokine array analysis (Proteome Profiler Mouse Cytokine Array Kit, Panel A, R&D System, Minneapolis, MN, USA), according to the manufacturer's instructions. Statistical analysis was performed using two‐way ANOVA followed by Fisher's *post hoc* test. The sequences of primer pairs are given in the supplementary material, Table S1 and further details of PCR in the supplementary material, Supplementary materials and methods.

### Laboratory analyses of serum and blood

Blood was collected from young and aging WT and KO mice (*n* = 7 per each group) under isoflurane overdose into BD Vacutainer tubes with sodium heparin for serum analysis or EDTA for routine complete blood count (Becton, Dickinson and Company, NJ, USA). Na^+^ and K^+^ ions, alkaline phosphatase (ALP), creatinine, cholesterol, triglyceride, and high‐ and low‐density lipoprotein (HDL, LDL) were measured with a COBAS 8000 analyzer (Roche Ltd, Rotkreuz, Switzerland), while blood count was measured with a Sysmex XN‐1000‐V Multispecies Hematology Analyzer (Sysmex Hungaria, Budapest, Hungary). Statistical analysis of serum parameters and blood count was performed using two‐way ANOVA followed by Fisher's or Bonferroni's *post hoc* test, respectively.

## Results

### Histopathological evaluation

Analysis of peripheral organs revealed more diffuse and more severe amyloidosis in the KO animals with progressive aging. First, we analyzed the number of animals with amyloidosis (Table 1). Signs in most organs had already appeared at 6 months in KO mice, whereas deposits appeared in WT animals typically at 15 months. The percentage of animals with deposits was markedly higher in KO groups than in their age‐matched WT mates. In WT mice, most severe deposits were observed in organs that showed some amyloidosis already at young ages, such as skin and intestines. In aging KO mice, liver, spleen, intestines, and thyroid gland were positive in all animals. Amyloid distribution in different organs is described below. The distribution of deposits was similar in both groups in general, with differences in the time of appearance and quantity. Altogether, amyloidosis manifested evidently earlier, progressed more rapidly with a higher degree of severity at older ages, and affected more KO individuals.

**Table 1:** Percentage of animals showing signs of amyloidosis

Organ	% of WT	% of WT	% of KO	% of KO
	3–12 months	13–24 months	3–12 months	13–24 months
Spleen	0	67	67	100
Esophagus	0	43	71	75
Kidney	0	33	57	86
Liver	0	17	29	100
Thyroid gland	0	25	60	100
Skin	14	67	57	88
Intestines	10	78	56	100
Heart	0	14	25	29
Lung	0	33	50	63
Stomach	0	60	43	50

#### Gastrointestinal tract

**Figure 1:**
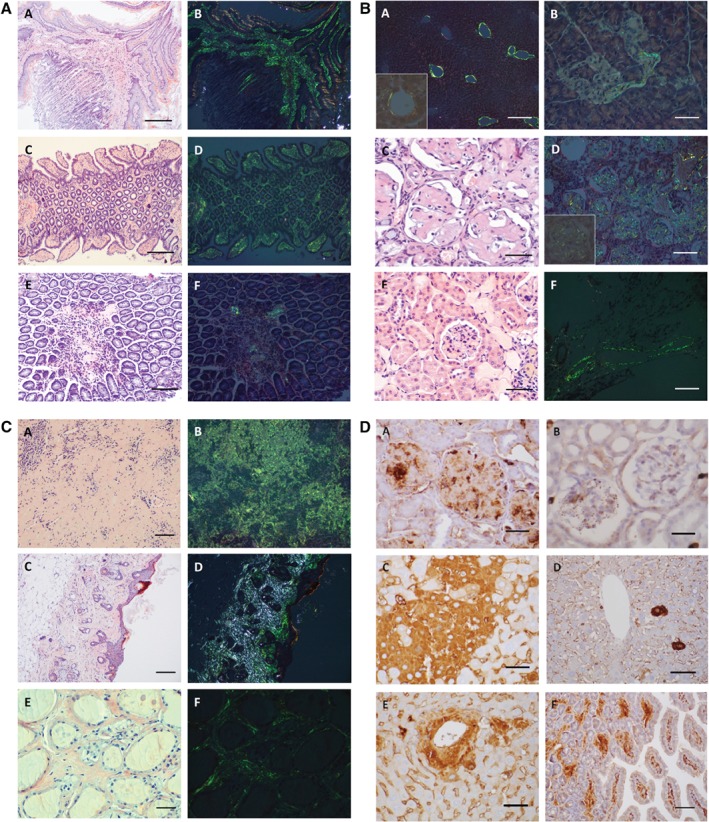
Representative photomicrographs of amyloid deposits in tissues from PACAP KO mice and of Apo‐AIV immunoreactivity in aged WT and PACAP KO mice. (Part A) Amyloid deposits in esophagus (A, B) and small intestine of a 16‐month‐old PACAP KO mouse (C, D), and WT of the same age (E, F). Congo red staining without polarizing light (A, C, E) showing red homogeneous amyloid deposits confirmed in the same locations of the same sections with polarizing light displaying apple‐green birefringence (B, D, F). Scale bar = 200 μm (A, B) or 170 μm (C–F). (Part B) Amyloid deposits in liver (A), pancreas (B), kidney (C, D), and a vein in adipose tissue (F) of a 16‐month‐old PACAP KO mouse and in the kidney of WT of the same age (E). Congo red staining without polarizing light (C, E) showing red homogeneous amyloid deposits, and apple‐green birefringence under polarizing light (A, B, D, F). Insets (A, D) show an enlarged area of a central vein in the liver (A) and a renal glomerulus (D). Scale bar = 350 μm (A), 80 μm (B, E), 30 μm (C), 100 μm (D), or 200 μm (F). (Part C) Amyloid deposits in spleen (A, B), skin (C, D), and thyroid gland (E, F) of a 16‐month‐old PACAP KO mouse. Congo red staining without polarizing light (A, C, E) showing red homogeneous deposits confirmed in the same sections with polarizing light displaying apple‐green birefringence (B, D, F). Scale bar = 250 μm (A–D) or 80 μm (E, F). (Part D) Apo‐AIV immunoreactivity in kidney glomeruli of aging KO (A) and aging WT (B), in degenerated hepatocytes in patches of an aging KO (C), and in single hepatocytes in an aging WT mouse (D) and around the central vein in an aging KO animal (E). Intestinal villi displaying immunoreactivity in an aging KO mouse (F). Scale bar = 30 μm (A, B), 70 μm (C–E) or 150 μm (F).

Esophageal lamina propria contained wide, filamentous, compact deposits in a linear distribution. In the stomach, mostly the interglandular connective tissue and vessels were affected (Figure 1A: A, B). Small intestinal villi showed the highest levels of deposits in the lamina propria and vessel walls, and interglandular connective tissue in small and large intestine was filled in severe cases with aging (Figure 1A: C–F). Salivary glands were unaffected; only occasional perivascular tissues and vessel walls were involved. In the liver, perivascular deposits were around portal vessels and the central vein. In sinusoidal walls, homogeneous amyloid deposits were seen around central veins (Figure 1B: A). Patches of degenerated hepatocytes were observed in KO mice, in contrast to WT mice, where only single cells were affected distal from central veins. Scattered degenerating hepatocytes, closely associated with deposits, were surrounded by amyloid‐containing macrophages. Intramural vascular deposits were found in the pancreas in a few animals (Figure 1B: B), and periductal connective tissue septa were affected, but islets, exocrine acini, and the gallbladder were unaffected.

#### Respiratory tract

In the trachea and larynx, amyloid was deposited in vessel walls. Under the parietal pleura, discrete focal deposits were observed in the lung, and intraparenchymal vessels contained amyloid. Alveolar cells and bronchial respiratory epithelium were unaffected.

#### Urogenital system

Deposits mainly affected kidney glomeruli; 90% were damaged by the amorphous deposits in severe cases in older KO animals, while amyloid was missing from tubules or interstitium (Figure 1B: C–E). No deposits were found in the uterus, vagina or ovaries of female mice, or in the testis, epididymis or seminal vesicles in males, only in peritesticular venous vessel walls.

#### Musculoskeletal and cardiovascular systems

In the skeletal muscle, small endomysial vessels were positive using Congo red. Joints or bones were unaffected. In the cardiac muscle, sub‐endocardial deposits were occasionally observed. In larger arteries and veins, deposits were mainly present both intramurally and perivascularly.

#### Immune system and skin

Only a few small patches were observed in WT spleens. Approximately 10% of the spleen was involved, without affecting the basic structure in young KO mice, while in older mice, approximately 65–70% of the spleen parenchyma was replaced by amyloid deposits in severe cases (Figure 1C: A, B). Deposits were seen in white and red pulps, and the sinusoid wall was infiltrated. Lymph nodes did not display positive deposits; only perinodular vessels contained some amyloid. No deposits were found in the thymus. In the skin, the main location of the deposits was the dermal papillary layer, continuous with the homogeneous mass in the connective tissue surrounding appendages (hair follicles and sebaceous and sweat glands) and vessel walls (Figure 1C: C, D).

#### Endocrine glands and nervous system

Diffuse deposits were observed surrounding thyroid follicles, which were free of deposits but often atrophic, resulting in fibrotic transformation of the parenchyma (Figure 1C: E, F). Other glands (adrenal, parathyroid, pituitary) displayed no positivity, similarly to the CNS, peripheral nerves, and eye (cornea, choroid layer, retina, sclera).

In several additional organs, perivascular connective tissue displayed some positivity. In addition, brown adipose tissue, typically present in mice in larger amounts, displayed deposits mainly in the connective tissue between adipocytes. In other organs, only sporadic, vessel wall‐related amyloid deposits were occasionally observed (Figure 1B: F).

**Figure 2:**
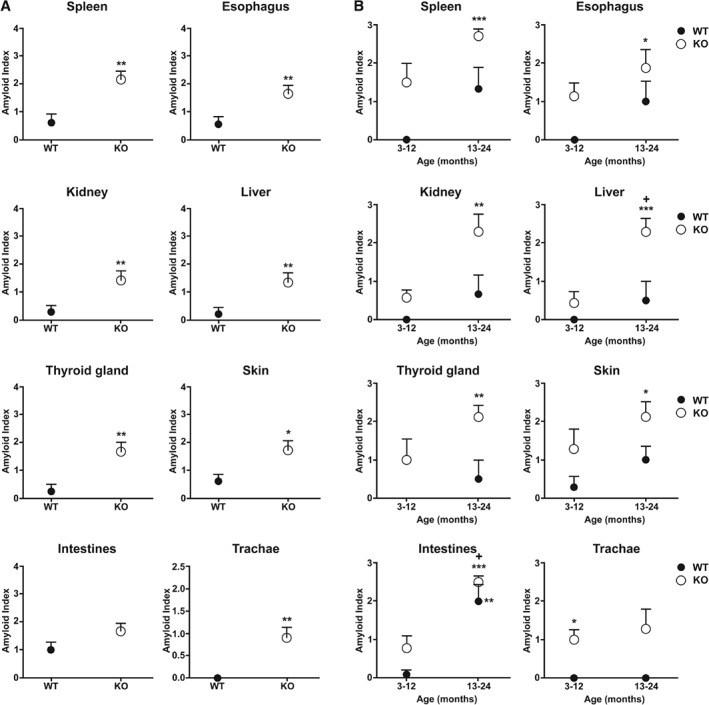
Amyloid index in young and old WT and PACAP KO mice. (A) Overall amyloid index in WT and PACAP KO mice in selected organs with massive amyloid deposits in old age. Mean ±SEM, where *p < 0.05 and **p < 0.01 versus WT mice. (B) Amyloid index in young (3–12 months) and aging (13–24 months) WT and PACAP KO mice in selected organs with massive amyloid deposits at older ages. Mean ±SEM, where *p < 0.05, **p < 0.01, and ***p < 0.001 versus young WT mice. ^+^ *p* < 0.05 versus young PACAP KO mice.

Statistical analysis of the amyloid index revealed a significant overall effect of PACAP in the spleen, esophagus, kidney, liver, thyroid gland, trachea, and skin (Figure 2A). The amyloid index in the young and older age groups also revealed significant differences between WT and KO groups. In most organs, the severity of amyloidosis was similar in aging WT and young KO mice, showing that amyloidosis had already reached senile levels at a young age as a result of a lack of PACAP (Figure 2B). Altogether, these data show that PACAP deficiency accelerates the appearance of senile systemic amyloidosis, resulting in pre‐senile degeneration with more generalized and more severe amyloid deposition.

### LMD‐based microproteomic analysis

**Figure 3:**
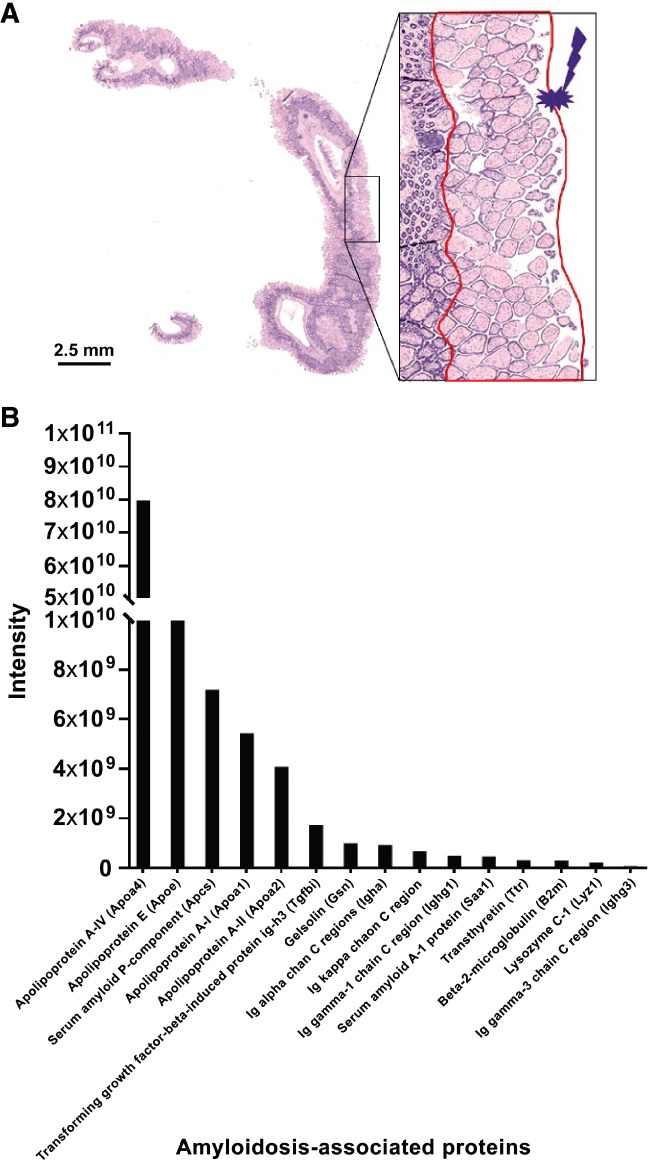
Characterization of the type of amyloidosis using laser microdissection‐based microproteomics. (A) Congo red‐stained section of the intestine of a PACAP KO mouse. The regions surrounded in red in the inset correspond to the laser‐microdissected one from a serial section. (B) Intensities of amyloidosis‐related proteins identified in the sample. The high intensity of Apoa4, Apoe, Apcs, and Apoa1 suggests that the amyloidosis type is AApoA4.

LMD‐based microproteomic analysis of amyloid deposits allowed confident identification of 13 235 peptides, corresponding to 2172 proteins. Fifteen known amyloidosis‐associated proteins were identified 10. These were, in order of intensity (Figure 3), Apo‐AIV, Apo‐E, serum amyloid P‐component, Apo‐AI, Apo‐AII, transforming growth factor‐beta‐induced protein ig‐h3, gelsolin, Ig alpha chain C region, Ig kappa chain C region, Ig gamma‐1 chain C region, serum amyloid A‐1 protein, transthyretin, beta‐2‐microglobulin, lysozyme C‐1, and Ig gamma‐3 chain C region (gene names: Apoa4, Apoe, Apcs, Apoa1, Apoa2, Tgfbi, Gsn, Igha, Ighg1, Saa1, Trt, B2m, Lyz1, Ighg3, respectively). The most abundant proteins were Apoa4, Apoe, Apcs, and Apoa1.

### MALDI IMS correlation with LMD‐based microproteomic dataset

**Figure 4:**
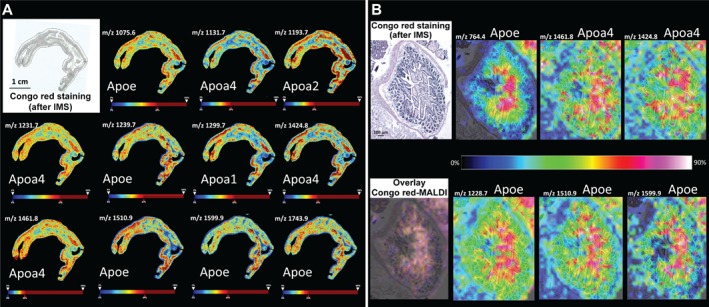
Mass spectrometric imaging of the amyloid deposits. (A) Correlation between Congo red and MALDI images of a mouse intestine tissue sample. MALDI IMS distribution of specific ion peptides identified as Apoe with *m/z* values 1075.6, 1239.7, 1510.9, 1599.9, and 1743.9; Apoa4 with *m/z* values 1131.7, 1231.7, 1424.8, and 1461.8; and Apoa1 with *m/z* value 1299.7 correlates with Congo red positive staining. (B) MALDI IMS distribution of the same ion peptides detected on a different mouse intestine tissue sample. A predictive peptide (*m/z* 764.4) for Apoe protein was also detected correlating exclusively with the amyloid deposits. Overlay of Congo red and MALDI images shows co‐localization of the different *m/z* species with extensive overlap with the amyloid deposits. MALDI IMS analysis was performed on the adjacent section to the Congo red staining section. The chromatic scale color bar indicates the relationship between the color of a peak displayed and the peak intensity in arbitrary units, with red and blue indicating the highest and lowest intensity color, respectively.

Sections with Congo red‐positive areas were examined under polarized light with digitally marked representative regions superimposed with MALDI data, and spectra profiles were compared between negative and positive areas in order to find discriminant peaks (Figure 4 and supplementary material, Figure S1). Peptide *m/z* values obtained from MALDI MSI were compared with peptides identified by LC–MS/MS to attribute IDs. We found 13 peptides identified by LC–MS/MS matching *m/z* values detected in deposits by MALDI IMS. Seven peptides were assigned to Apoe, four to Apoa4, one to Apoa1, and one to Apoa2. To evaluate the difference of all *m/z* peptide values through the two regions, the receiver operating characteristic (ROC) test was performed and the area under the ROC curve (AUC) measured was greater than 0.9 for all peptides except for *m/z* 1239.7 and 1743.9, where the AUC was more than 0.7.

### Immunohistochemistry for Apo‐AIV and molecular analysis

As Apoa4 was identified as the main amyloidosis‐related protein in the deposits by LC–MS/MS, we confirmed the presence of this apolipoprotein by immunohistochemistry in the organs where the most massive amyloidosis was found. We found Apo‐AIV‐positive areas in the examined organs corresponding to the distribution of Congo red‐positive regions in aging animals (Figure 1D). In KO mice, Apo‐AIV immunopositivity was stronger and more widespread than in WT mice, in accordance with the histopathological observations.

**Figure 5:**
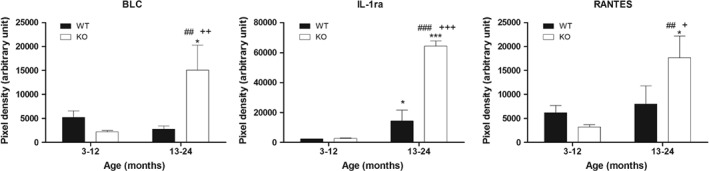
Results from cytokine array analysis. Plots show pixel densities of BLC (B‐lymphocyte chemoattractant), IL‐1ra (interleukin‐1 receptor antagonist), and RANTES (regulated on activation, normal T‐cell expressed and secreted) in young and aging wild‐type (WT) and PACAP knockout (KO) kidneys. Mean ±SEM. *p < 0.05, ***p < 0.001 versus WT 3–12; ^##^ *p* < 0.01, ^###^ *p* < 0.001 versus KO 3–12; ^+^ *p* < 0.05, ^++^ *p* < 0.01, ^+++^ *p* < 0.001 versus WT 13–24.

The balance in the expression of mRNA encoding amyloid specific proteins identified with MS in the intestine was disturbed in the absence of PACAP, with some showing stronger, while others weaker expression in the KO samples than in the WT ones (supplementary material, Figure S2). The results of the cytokine array revealed dramatically increased levels of several cytokines in the aging KO samples compared with the other groups. Of those, the most marked changes were observed in BLC (B lymphocyte chemoattractant), IL‐1ra (interleukin‐1 receptor antagonist), and RANTES (regulated on activation, normal T‐cell expressed and secreted) (Figure 5).

### Serum and blood analyses

Serum analysis revealed no difference in Na^+^ or K^+^ levels. Among the parameters referring to liver and kidney functional deficit, ALP and creatinine were examined, respectively. While there was no difference in ALP levels, serum creatinine levels showed a marked, significant increase in aging versus young PACAP KO mice in contrast to WT animals, where there was no age‐related increase. Lipid parameters (HDL, LDL, total cholesterol) showed slight but not statistically significant increases in aging PACAP KO animals (supplementary material, Figure S3). The complete blood count showed no significant alterations between the examined groups.

## Discussion

The increased vulnerability to stressors of animals lacking PACAP and its well‐known cytoprotective effects support the hypothesis that PACAP deficiency mimics aspects of age‐related pathophysiological changes. Here, we have described the premature appearance of severe senile systemic amyloidosis in mice lacking endogenous PACAP. We showed with mass spectrometry‐based proteomic analysis, confirmed by immunohistochemistry, that apolipoprotein‐AIV was the main amyloid protein in the deposits with several accompanying ones. These findings indicate that in the absence of endogenous PACAP, accelerated age‐related degenerative changes occur, pointing to the important role of PACAP in aging‐induced systemic processes.

It is still not entirely understood why deposits are formed at an older age and why only in some tissues 38. Several animal models have been proposed to study age‐related systemic amyloidosis, since spontaneous amyloidosis has long been known to occur in various strains of inbred mice 39. A study in different mice 5 described spontaneous systemic senile amyloidosis in 75% of strains. This was confirmed in a 15‐year survey, where a higher incidence of amyloidosis was found in CD1 mice than in others 40. In our CD1 mice, we found no deposits during the first year in WTs (only in sporadic skin and intestinal spots), but already at early ages in some PACAP KO individuals. Our results, showing a few individuals with slight amyloidosis at 6 months, are also in accordance with the description of spontaneous amyloidosis in aging inbred mice 8. The observation that the degree of fibril formation showed no gender difference is also in agreement with earlier findings of CD1 and other strains 33, 40, 41. Here, we have described a type of age‐related systemic amyloidosis with mainly kidney, spleen, liver, skin, thyroid, intestine, stomach, trachea, and esophagus involvement. The severe kidney involvement is also reflected in higher creatinine levels, indicating functional disturbance. While most forms of systemic amyloidosis affect joints and periarticular tissues 42, we did not observe articular amyloid deposits. Our observation that the CNS and peripheral nerves lacked deposits is in accord with earlier findings in systemic amyloidosis 8, 43, 44.

It is now known that amyloid deposits contain several distinct main and accompanying minor components 10, 45 that can be structurally unrelated and can be deposited in different locations in conglomerates or in distinct foci 46, 47. Proteomic approaches have identified the composition of the deposits 5, 48, 49, 50, 51, 52, 53, with Apoe, serum amyloid P component, Apoa4, and Apoa1 present in almost all amyloidosis types as incorporated proteins 10. Of these, the first three are considered specific amyloidosis biomarkers. We used LC–MS/MS‐based proteomics, adequate for the identification of amyloidosis‐associated proteins and the subsequent classification of amyloidosis subtypes 10, 34, 35, 36, 48, 52, 54, 55. In the present context, deposits could be found in large quantities within different organs. We could identify with high confidence 15 amyloidosis‐related proteins, with Apoa4 having the highest abundance 10, 48. It is noteworthy that non‐amyloidosis‐associated proteins reported in human cases of AApoAIV amyloidosis such as vitronectin, serum albumin, clusterin, collagen alpha‐2 (I) chain, collagen alpha‐1 (I) chain, and actin 48 were also found in our samples. The combined analysis of LMD‐based microproteomic and MALDI IMS resulted in a more comprehensive characterization, similarly to previous descriptions in renal biopsies 56. By matching the peptide masses identified by LC–MS/MS with MALDI IMS, 13 peptides related to Apoa4 (*n*_peptides_ = 4), Apoe (*n*_peptides_ = 7), Apoa1 (*n*_peptides_ = 1), and Apoa2 (*n*_peptides_ = 1) were detected exclusively in amyloid regions, in excellent concordance with the Congo red‐positive areas.

The systemic amyloidosis‐associated protein described here was predominantly Apo‐AIV, confirmed by Apo‐AIV immunohistochemistry. We could also detect mRNA for the proteins identified by MS in the same tissues, but the balance of the different amyloidogenic protein mRNAs was disturbed, possibly indicating a pathological feedback for production due to the local deposits and/or disturbed synthesis. Apolipoproteins play a role in lipid metabolism and transport 57 with susceptibility to form fibrils, possibly due to their intrinsic flexibility 46. Considering the role of apolipoproteins in lipid metabolism, we ran a laboratory test for the main lipid parameters, but found no significant differences. This indicates no major disturbances in lipid metabolism in general, but raises the possibility of some other mechanisms leading to apolipoprotein deposits in KO tissues. Apolipoproteins have long been associated with fibril formation in cerebral and systemic amyloidosis 58. Among apolipoproteins, Apo‐AIV shows age‐related decline (which we could also detect in KO mice) and has the highest sensitivity to denaturation and vulnerability to environmental disturbances 46. Recent reports using proteomics‐based assays have shown a more widespread occurrence of AApoAIV amyloidosis than previously thought 48, 50. Apo‐E can also be consistently detected in deposits 59, 60, and Apo‐AI has long been associated with systemic amyloidosis with different localizations 51, 61. AApoAIV‐ and AApoE‐dominating systemic amyloidosis is known in inbred foxes with mainly kidney, spleen, oral cavity, and vascular involvement 62. Apo‐AII is also known to form senile fibrils 33, 44, 63 including inbred mouse strains 8. Serum amyloid P component, a constituent in all amyloid deposits 10, 63, 64, was also found in our samples. Although its role is controversial, i.e. both anti‐amyloidogenic and pro‐amyloidogenic effects have been described 65, its presence is usually attributed to its role in stabilizing the fibrils, thereby preventing proteolytic degradation 66, 67. A recent study has suggested that in addition to the most common proteins, serum amyloid P component, Apo‐E, and Apo‐AIV, the amyloid proteomic signature should also include vitronectin and clusterin as co‐deposited proteins 53. We could also detect other proteins associated with amyloid formation, such as gelsolin, transthyretin, Ig kappa and gamma‐1 chain C region, serum amyloid A1, beta2 microglobulin, and lysozyme C1. As these proteins were detected with relatively low intensities, they do not seem to be major amyloid constituents in PACAP KO mice.

Good animal models are important in studying diseases of protein misfolding, as it is still unknown why the same protein becomes fibrillogenic with age and why only in some tissues 38. It has been assumed that age‐related forms of amyloidosis reflect chronic inflammatory states, high concentrations of amyloidogenic proteins, and a long latent period of seed formation. In our study, chronic inflammation is excluded, due to a lack of histological signs of generalized inflammation or changes in blood cell counts. However, we detected an altered cytokine profile, especially in aging KO mice. These data indicate a disturbed balance of pro‐ and anti‐inflammatory cytokines and may lead to a shift in the cytokine profile towards a pro‐inflammatory milieu, possibly creating a more favorable microenvironment for amyloid deposition in mice lacking PACAP. However, the altered cytokine profile might as well be the consequence of amyloid deposition, leading to upregulation of both pro‐ and anti‐inflammatory cytokines. Earlier studies found differences in the proteome of PACAP KO mice, also leading to a disturbed energy balance, which is possibly further disturbed in aging 28. Alterations in cytokine expression have been described earlier in several experimental paradigms after PACAP treatment and have been suggested as an aggravating factor in slower regeneration capacity of KO mice 68, 69.

**Figure 6:**
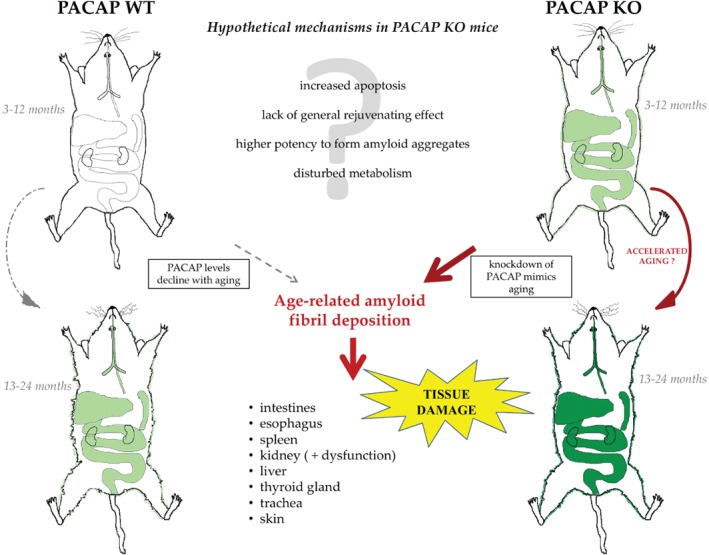
Graphical summary of the main findings showing that lack of PACAP accelerates and aggravates the appearance of senile systemic amyloidosis. Green color indicates the appearance of amyloid deposits. Only organs with deposits are depicted, with darker colors indicating more severe amyloidosis.

Age‐related degenerative diseases are increasing, and the problem goes far beyond the two best‐known diseases, Alzheimer's and Parkinson's, with protein misfolding in the background 70. PACAP levels decline with age in humans and experimental animals 71, as shown in rhesus monkey and post‐mortem human brains of Alzheimer's patients and in cognitive decline preceding the disease 22, 23, 72, 73, with an association between PACAP levels and disease scores. The few data available to date indicate that PACAP deficiency accelerates aging. Aging KO animals display higher levels of oxidative stress 26, an earlier appearance of retinal degeneration 27, and decreased tear secretion with increased corneal keratinization 74. In the present study, we have described accelerated systemic senile amyloidosis in PACAP‐deficient mice, which might indicate an early aging phenomenon in this strain. Thus, PACAP KO mice could serve as a model of accelerated aging with human relevance, similarly to the widely used senescence‐accelerated mouse, which also displays spontaneous age‐associated amyloidosis 41, 74, 75. Exogenous PACAP can compensate the lack of PACAP, as shown in several acute injury models in KO mice. Among others, PACAP administration could reduce the toxic effects of ethanol or oxidative stress in cerebellar neurons isolated from PACAP KO mice 76. Exogenous PACAP addition could also attenuate the increased ischemia‐induced lesions in KO retinas 77. Furthermore, Ohtaki *et al* found a greater infarct volume and worse behavioral deficits in hetero‐ and homozygous PACAP‐deficient mice than in WT ones. However, both infarct volume and neurological deficit scores were improved by PACAP injection 69. The effectivity of factors slowing down misfolded fibril formation and their aggregation decreases, while amyloidogenic mechanisms are accelerated with aging, especially in cases of genetic susceptibility 70, 78, 79. Alterations in metabolic enzymes might partially account for the decreased antioxidant and detoxifying capacity of PACAP KO mice 2, 28. The disturbed balance in homeostatic molecules, inflammatory factors, and metabolic enzymes may be compensated at a young age under normal conditions, while they create an unfavorable milieu for increased vulnerability in cases of injury and aging‐related processes. Several other properties of PACAP might be involved in cytoprotection in amyloidosis; for example, PACAP prevents toxic amyloid‐beta formation 80, 81, rescues cells from amyloid toxicity 73, 82, acts against increased apoptosis associated with amyloidosis 83, 84, and has a unique potency to bind to negatively charged glycosaminoglycans 85, 86, which in turn co‐accumulate with amyloid 87. It is proposed that PACAP plays a role in the complex cellular interactions that lead to tissue destruction in amyloidosis 88 and that the lack of the general rejuvenating effect of PACAP accelerates amyloid formation (Figure 6). PACAP‐deficient mice, with their higher vulnerability to noxious stimuli, resemble aging cells with vulnerability to cell death 70. The physiological or pathological decline of PACAP leads to an increased susceptibility for degenerative processes in the elderly, and thus to accelerated age‐related diseases. Our observations point to a multifaceted role of PACAP in aging and age‐related degenerative processes and could initiate further studies on the role of PACAP in aging.

## Author contributions statement

DR, AJ, AT, and ZN prepared tissues. DR, AB, SB, JK, and MK performed histopathological analysis. AJ, AT, and JS performed statistical analysis of the histological scores. RL, RC, JK, and GM performed mass spectrometry analysis. HH provided the gene‐deficient animals. BDF maintained the gene‐deficient animal colony and performed PCR genotyping. KK, AJ, and JS performed cytokine array and western blot measurements. AM, AJ, and JS performed laboratory tests of serum samples; TJ performed PCR analysis. DR and AB conceptualized the experiments. DR, AB, AJ, RC, RL, and AT prepared the manuscript.

**SUPPLEMENTARY MATERIAL ONLINE**

**Supplementary materials and methods**

**Figure S1.** Correlation between Congo red and the MALDI image of *m/z* 1228.7 from the mouse intestine tissue section presented in Figure 4

**Figure S2.** Representative data of three independent RT‐PCR reactions of amyloid specific protein mRNAs identified with mass spectrometry of aging WT and KO mice

**Figure S3.** Main laboratory serum parameters in young and old WT and PACAP KO mice

**Table S1.** Nucleotide sequences, amplification sites, GenBank accession numbers, amplimer sizes, and PCR reaction conditions for each mouse primer pair are shown

## Supporting information


**Supplementary materials and methods**

**Figure S1. Correlation between Congo‐red and the MALDI image of m/z 1228.7 from the mouse intestine tissue section presented in Figure 4.** MALDI IMS distribution the ion peptide identified as Apoe with m/z value 1228.7 in a section of intestine with amyloidosis (right). Same section stained with Congo red after MALDI IMS (left); this Congo red panel is the same as shown in Figure 4.
**Figure S2. Representative data of three independent RT‐PCR reactions of amyloid specific protein mRNAs identified with mass spectrometry of aging WT and KO mice.** Optical density of signals was measured and results were normalized to the optical density of controls. *Gapdh* was used as internal control. Numbers below the bands represent relative integrated densities of signals. The mRNA expression of *Gsn*, *Lyz1*, *Apcs* and *Saa1* was augmented in the KO mice. However, that of *B2m*, *Ighg1* and *Igκ cC* did not alter compared with WT animals. The mRNA expression of *Ttr* increased the most prominently and appeared with a three times stronger lane than in WT samples. Opposite to the protein level, expression of *Apoa1* and *Apoa4* mRNA was decreased in PACAP KO mice, while that of *Apoe* was increased. However, mRNA expression of *Apoa2*, the accumulation of which has been detected in Alzheimer disease, could not be shown. *p<0.05 compared to respective controls.
**Figure S3. Main laboratory serum parameters in young and old WT and PACAP KO mice.** Values are given as mean±SEM, where *p<0.05 versus young mice.

**Table S1.** Nucleotide sequences, amplification sites, GenBank accession numbers, amplimer sizes and PCR reaction conditions for each mouse primer pair are shown

## References

[1] Reglodi D , Kiss P , Lubics A , *et al* Review on the protective effects of PACAP in models of neurodegenerative diseases *in vitro* and *in vivo* . Curr Pharm Des 2011; 17 **:** 962–972. 21524257 10.2174/138161211795589355

[2] Reglodi D , Kiss P , Szabadfi K , *et al* PACAP is an endogenous protective factor – insights from PACAP‐deficient mice. J Mol Neurosci 2012; 48 **:** 482–492. 22528455 10.1007/s12031-012-9762-0

[3] Aguzzi A , O'Connor T . Protein aggregation diseases: pathogenicity and therapeutic perspectives. Nat Rev Drug Discov 2010; 9 **:** 237–248. 20190788 10.1038/nrd3050

[4] Buxbaum JN , Linke RP . A molecular history of the amyloidoses. J Mol Biol 2012; 421 **:** 142–159. 22321796 10.1016/j.jmb.2012.01.024

[5] Lavatelli F , Perlman DH , Spencer B , *et al* Amyloidogenic and associated proteins in systemic amyloidosis proteome of adipose tissue. Mol Cell Proteomics 2008; 7 **:** 1570–1583. 18474516 10.1074/mcp.M700545-MCP200 PMC2494907

[6] Chiti F , Dobson CM . Protein misfolding, amyloid formation, and human disease: a summary of progress over the last decade. Annu Rev Biochem 2017; 86 **:** 27–68. 28498720 10.1146/annurev-biochem-061516-045115

[7] Falk RH , Comenzo RL , Skinner M . The systemic amyloidoses. N Engl J Med 1997; 337 **:** 898–909. 9302305 10.1056/NEJM199709253371306

[8] Higuchi K , Naiki H , Kitagawa K , *et al* Mouse senile amyloidosis . ASSAM amyloidosis in mice presents universally as a systemic age‐associated amyloidosis. Virchows Arch B Cell Pathol Incl Mol Pathol 1991; 60 **:** 231–238. 1681611

[9] Cui D , Hoshii Y , Takahashi M , *et al* An immunohistochemical study of amyloid P component, apolipoprotein E and ubiquitin in human and murine amyloidoses. Pathol Int 1998; 48 **:** 362–367. 9704343 10.1111/j.1440-1827.1998.tb03919.x

[10] Dogan A . Amyloidosis: insights from proteomics. Annu Rev Pathol 2017; 12 **:** 277–304. 27959636 10.1146/annurev-pathol-052016-100200

[11] Noda S , So M , Adachi M , *et al* Thioflavin T‐silent denaturation intermediates support the main‐chain‐dominated architecture of amyloid fibrils. Biochemistry 2016; 55 **:** 3937–3948. 27345358 10.1021/acs.biochem.6b00231

[12] Martin EB , Williams A , Richey T , *et al* Comparative evaluation of p5 + 14 with SAP and peptide p5 by dual‐energy SPECT imaging of mice with AA amyloidosis. Sci Rep 2016; 6 **:** 22695. 10.1038/srep22695 PMC4776142 26936002

[13] Kisilevsky R , Fraser PE . A beta amyloidogenesis: unique, or variation on a systemic theme? Crit Rev Biochem Mol Biol 1997; 32 **:** 361–404. 9383610 10.3109/10409239709082674

[14] Fosgerau K , Hoffmann T . Peptide therapeutics: current status and future directions. Drug Discov Today 2015; 20 **:** 122–128. 25450771 10.1016/j.drudis.2014.10.003

[15] Brahmachari S , Paul A , Segal D , *et al* Inhibition of amyloid oligomerization into different supramolecular architectures by small molecules: mechanistic insights and design rules. Future Med Chem 2017; 9 **:** 797–810. 28485623 10.4155/fmc-2017-0026

[16] Ogren SO , Kuteeva E , Elvander‐Tottie E , *et al* Neuropeptides in learning and memory processes with focus on galanin. Eur J Pharmacol 2010; 626 **:** 9–17. 19837050 10.1016/j.ejphar.2009.09.070

[17] Somogyvari‐Vigh A , Reglodi D . Pituitary adenylate cyclase activating polypeptide: a potential neuroprotective peptide. Curr Pharm Des 2004; 10 **:** 2861–2889. 15379674 10.2174/1381612043383548

[18] Vaudry D , Falluel‐Morel A , Bourgault S , *et al* Pituitary adenylate cyclase‐activating polypeptide and its receptors: 20 years after the discovery. Pharmacol Rev 2009; 61 **:** 283–357. 19805477 10.1124/pr.109.001370

[19] Reglodi D, Tamas A (Eds). Pituitary Adenylate Cyclase Activating Polypeptide – PACAP. Current Topics in Neurotoxicity, Vol 11. Springer Nature: New York, 2016.

[20] Abad C , Waschek JA . Immunomodulatory roles of VIP and PACAP in models of multiple sclerosis. Curr Pharm Des 2011; 17 **:** 1025–1035. 21524252 10.2174/138161211795589364

[21] Seaborn T , Masmoudi‐Kouli O , Fournier A , *et al* Protective effects of pituitary adenylate cyclase‐activating polypeptide (PACAP) against apoptosis. Curr Pharm Des 2011; 17 **:** 204–214. 21348830 10.2174/138161211795049679

[22] Han P , Caselli RJ , Baxter L , *et al* Association of pituitary adenylate cyclase‐activating polypeptide with cognitive decline in mild cognitive impairment due to Alzheimer disease. JAMA Neurol 2015; 72 **:** 333–339. 25599520 10.1001/jamaneurol.2014.3625 PMC5924703

[23] Han P , Nielsen M , Song M , *et al* The impact of aging on brain pituitary adenylate cyclase activating polypeptide, pathology and cognition in mice and rhesus macaques. Front Aging Neurosci 2017; 9 **:** 180. 28659785 10.3389/fnagi.2017.00180 PMC5467357

[24] Banki E , Sosnowska D , Tucsek Z , *et al* Age‐related decline of autocrine pituitary adenylate cyclase‐activating polypeptide impairs angiogenic capacity of rat cerebromicrovascular endothelial cells. J Gerontol A Biol Sci Med Sci 2015; 70 **:** 665–674. 25136000 10.1093/gerona/glu116 PMC4447801

[25] Tripathy D , Sanchez A , Yin X , *et al* Age‐related decrease in cerebrovascular‐derived neuroprotective proteins: effect of acetaminophen. Microvasc Res 2012; 84 **:** 278–285. 22944728 10.1016/j.mvr.2012.08.004 PMC3483357

[26] Ohtaki H , Satoh A , Nakamachi T , *et al* Regulation of oxidative stress by pituitary adenylate cyclase‐activating polypeptide (PACAP) mediated by PACAP receptor. J Mol Neurosci 2010; 42 **:** 397–403. 20387010 10.1007/s12031-010-9350-0

[27] Kovacs‐Valasek A , Szabadfi K , Denes V , *et al* Accelerated retinal aging in PACAP knock‐out mice. Neuroscience 2017; 348 **:** 1–10. 28215987 10.1016/j.neuroscience.2017.02.003

[28] Maasz G , Pirger Z , Reglodi D , *et al* Comparative protein composition of the brains of PACAP‐deficient mice using mass spectrometry‐based proteomic analysis. J Mol Neurosci 2014; 54 **:** 310–319. 24643519 10.1007/s12031-014-0264-0

[29] Hashimoto H , Shintani N , Tanaka K , *et al* Altered psychomotor behaviors in mice lacking pituitary adenylate cyclase‐activating polypeptide (PACAP). Proc Natl Acad Sci U S A 2001; 98 **:** 13355–13360. 11687615 10.1073/pnas.231094498 PMC60875

[30] Farkas J , Kovacs L , Gaspar L , *et al* Construct and face validity of a new model for the three‐hit theory of depression using PACAP mutant mice on CD1 background. Neuroscience 2017; 354 **:** 11–29. 28450265 10.1016/j.neuroscience.2017.04.019

[31] Linke RP . Highly sensitive diagnosis of amyloid and various amyloid syndromes using Congo red fluorescence. Virchows Arch 2000; 436 **:** 439–448. 10881737 10.1007/s004280050471

[32] Seymour JF , Lieschke GJ , Grail D , *et al* Mice lacking both granulocyte colony‐stimulating factor (CSF) and granulocyte‐macrophage CSF have impaired reproductive capacity, perturbed neonatal granulopoiesis, lung disease, amyloidosis, and reduced long‐term survival. Blood 1997; 90 **:** 3037–3049. 9376584

[33] Li L , Sawashita J , Ding X , *et al* Caloric restriction reduces the systemic progression of mouse AApoAII amyloidosis. PLoS One 2017; 12 **:** e0172402. 10.1371/journal.pone.0172402 PMC5321440 28225824

[34] Longuespee R , Alberts D , Pottier C , *et al* A laser microdissection‐based workflow for FFPE tissue microproteomics: important considerations for small sample processing. Methods 2016; 104 **:** 154–162. 26690073 10.1016/j.ymeth.2015.12.008

[35] Herfs M , Longuespee R , Quick CM , *et al* Proteomic signatures reveal a dualistic and clinically relevant classification of anal canal carcinoma. J Pathol 2017; 241 **:** 522–533. 27976366 10.1002/path.4858

[36] Longuespée R , Casadonte R , Kriegsmann M , *et al* Proteomic investigation of human cystic echinococcosis in the liver. Mol Biochem Parasitol 2017; 211 **:** 9–14. 27986452 10.1016/j.molbiopara.2016.12.002

[37] Longuespee R , Baiwir D , Mazzucchelli G , *et al* Laser microdissection‐based microproteomics of formalin‐fixed and paraffin‐embedded (FFPE) tissues. Methods Mol Biol 2018; 1723 **:** 19–31. 29344853 10.1007/978-1-4939-7558-7_2

[38] Buxbaum JN . Animal models of human amyloidoses: are transgenic mice worth the time and trouble? FEBS Lett 2009; 583 **:** 2663–2673. 19627988 10.1016/j.febslet.2009.07.031 PMC2749068

[39] Higuchi K , Matsumura A , Honma A , *et al* Systemic senile amyloid in senescence‐accelerated mice. A unique fibril protein demonstrated in tissues from various organs by the unlabeled immunoperoxidase method. Lab Invest 1983; 48 **:** 231–240. 6337302

[40] Majeed SK . Survey on spontaneous systemic amyloidosis in aging mice. Arzneimittelforschung 1993; 43 **:** 170–178. 8457241

[41] Takeshita S , Hosokawa M , Irino M , *et al* Spontaneous age‐associated amyloidosis in senescence‐accelerated mouse (SAM). Mech Ageing Dev 1982; 20 **:** 13–23. 7176700 10.1016/0047-6374(82)90070-7

[42] Perfetto F , Moggi‐Pignone A , Livi R , *et al* Systemic amyloidosis: a challenge for the rheumatologist. Nat Rev Rheumatol 2010; 6 **:** 417–429. 20531382 10.1038/nrrheum.2010.84

[43] Brayton C , Fox JG , Barthold SW , *et al* Spontaneous diseases in commonly used mouse strains In The Mouse in Biomedical Research. Academic Press, New York, USA, 2006; 623–717.

[44] Wang Y , Sawashita J , Qian J , *et al* ApoA‐I deficiency in mice is associated with redistribution of apoA‐II and aggravated AApoAII amyloidosis. J Lipid Res 2011; 52 **:** 1461–1470. 21622630 10.1194/jlr.M013235 PMC3133974

[45] Woldemeskel M . A concise review of amyloidosis in animals. Vet Med Int 2012; 2012 **:** 427296. 10.1155/2012/427296 PMC3329740 22577608

[46] Bergstrom J , Murphy CL , Weiss DT , *et al* Two different types of amyloid deposits – apolipoprotein A‐IV and transthyretin – in a patient with systemic amyloidosis. Lab Invest 2004; 84 **:** 981–988. 15146166 10.1038/labinvest.3700124

[47] Tashiro F , Yi S , Wakasugi S , *et al* Role of serum amyloid P component for systemic amyloidosis in transgenic mice carrying human mutant transthyretin gene. Gerontology 1991; 37 **(** suppl 1): 56–62. 1937069 10.1159/000213298

[48] Dasari S , Amin MS , Kurtin PJ , *et al* Clinical, biopsy, and mass spectrometry characteristics of renal apolipoprotein A‐IV amyloidosis. Kidney Int 2016; 90 **:** 658–664. 27262366 10.1016/j.kint.2016.04.003

[49] Brambilla F , Lavatelli F , Di Silvestre D , *et al* Reliable typing of systemic amyloidoses through proteomic analysis of subcutaneous adipose tissue. Blood 2012; 119 **:** 1844–1847. 21917755 10.1182/blood-2011-07-365510

[50] Obici L , Nuvolone M , Merlini G . Expanding the spectrum of systemic amyloid diseases: a new hint from the kidney. Kidney Int 2016; 90 **:** 479–481. 27521110 10.1016/j.kint.2016.05.029

[51] Solomon A , Murphy CL , Kestler D , *et al* Amyloid contained in the knee joint meniscus is formed from apolipoprotein A‐I. Arthritis Rheum 2006; 54 **:** 3545–3550. 17075859 10.1002/art.22201

[52] Vrana JA , Gamez JD , Madden BJ , *et al* Classification of amyloidosis by laser microdissection and mass spectrometry‐based proteomic analysis in clinical biopsy specimens. Blood 2009; 114 **:** 4957–4959. 19797517 10.1182/blood-2009-07-230722

[53] Mollee P , Boros S , Loo D , *et al* Implementation and evaluation of amyloidosis subtyping by laser‐capture microdissection and tandem mass spectrometry. Clin Proteomics 2016; 13 **:** 30. 27795698 10.1186/s12014-016-9133-x PMC5081679

[54] Sethi S , Theis JD , Leung N , *et al* Mass spectrometry‐based proteomic diagnosis of renal immunoglobulin heavy chain amyloidosis. Clin J Am Soc Nephrol 2010; 5 **:** 2180–2187. 20876678 10.2215/CJN.02890310 PMC2994078

[55] Sethi S , Vrana JA , Theis JD , *et al* Laser microdissection and mass spectrometry‐based proteomics aids the diagnosis and typing of renal amyloidosis. Kidney Int 2012; 82 **:** 226–234. 22495291 10.1038/ki.2012.108 PMC3388518

[56] Casadonte R , Kriegsmann M , Deininger SO , *et al* Imaging mass spectrometry analysis of renal amyloidosis biopsies reveals protein co‐localization with amyloid deposits. Anal Bioanal Chem 2015; 407 **:** 5323–5331. 25935672 10.1007/s00216-015-8689-z

[57] Kohan AB , Wang F , Lo CM , *et al* ApoA‐IV: current and emerging roles in intestinal lipid metabolism, glucose homeostasis, and satiety. Am J Physiol Gastrointest Liver Physiol 2015; 308 **:** G472–G481. 25591862 10.1152/ajpgi.00098.2014 PMC4360046

[58] Kindy MS , Rader DJ . Reduction in amyloid A amyloid formation in apolipoprotein‐E‐deficient mice. Am J Pathol 1998; 152 **:** 1387–1395. 9588907 PMC1858574

[59] Kindy MS , King AR , Perry G , *et al* Association of apolipoprotein E with murine amyloid A protein amyloid. Lab Invest 1995; 73 **:** 469–475. 7474917

[60] Yamada T , Someya T , Fujita S . Immunotargeting of apolipoprotein E in amyloid: an initial trial in mice. Ann Clin Lab Sci 2009; 39 **:** 134–137. 19429798

[61] Arciello A , Piccoli R , Monti DM . Apolipoprotein A‐I: the dual face of a protein. FEBS Lett 2016; 590 **:** 4171–4179. 27790714 10.1002/1873-3468.12468

[62] Gaffney PM , Imai DM , Clifford DL , *et al* Proteomic analysis of highly prevalent amyloid A amyloidosis endemic to endangered island foxes. PLoS One 2014; 9 **:** e113765. 10.1371/journal.pone.0113765 PMC4245998 25429466

[63] Qian J , Yan J , Ge F , *et al* Mouse senile amyloid fibrils deposited in skeletal muscle exhibit amyloidosis‐enhancing activity. PLoS Pathog 2010; 6 **:** e1000914. 10.1371/journal.ppat.1000914 PMC2873911 20502680

[64] Usui I , Kawano H , Ito S , *et al* Homozygous serum amyloid P component‐deficiency does not enhance regression of AA amyloid deposits. Amyloid 2001; 8 **:** 101–104. 11409030 10.3109/13506120109007351

[65] Ozawa D , Nomura R , Mangione PP , *et al* Antiamyloidogenic and proamyloidogenic chaperone effects of C‐reactive protein and serum amyloid P component. Amyloid 2017; 24 **:** 28–29. 28434325 10.1080/13506129.2017.1295943

[66] Botto M , Hawkins PN , Bickerstaff MC , *et al* Amyloid deposition is delayed in mice with targeted deletion of the serum amyloid P component gene. Nat Med 1997; 3 **:** 855–859. 9256275 10.1038/nm0897-855

[67] Togashi S , Lim SK , Kawano H , *et al* Serum amyloid P component enhances induction of murine amyloidosis. Lab Invest 1997; 77 **:** 525–531. 9389795

[68] Watson MB , Nobuta H , Abad C , *et al* PACAP deficiency sensitizes nigrostriatal dopaminergic neurons to paraquat‐induced damage and modulates central and peripheral inflammatory activation in mice. Neuroscience 2013; 240 **:** 277–286. 23500093 10.1016/j.neuroscience.2013.03.002 PMC3637876

[69] Ohtaki H , Nakamachi T , Dohi K , *et al* Pituitary adenylate cyclase‐activating polypeptide (PACAP) decreases ischemic neuronal cell death in association with IL‐6. Proc Natl Acad Sci U S A 2006; 103 **:** 7488–7493. 16651528 10.1073/pnas.0600375103 PMC1464366

[70] Stroo E , Koopman M , Nollen EA , *et al* Cellular regulation of amyloid formation in aging and disease. Front Neurosci 2017; 11 **:** 64. 28261044 10.3389/fnins.2017.00064 PMC5306383

[71] Reglodi D , Helyes Z , Nemeth J , *et al* PACAP as a potential biomarker – alterations of PACAP levels in human physiological and pathological conditions In Pituitary Adenylate Cyclase Activating Polypeptide – PACAP. Current Topics in Neurotoxicity, Reglodi D, Tamas A (eds), Vol 11 Springer Nature: New York, 2016; Ch 48, 815–832.

[72] Han P , Liang W , Baxter LC , *et al* Pituitary adenylate cyclase‐activating polypeptide is reduced in Alzheimer disease. Neurology 2014; 82 **:** 1724–1728. 24719484 10.1212/WNL.0000000000000417 PMC4032204

[73] Han P , Tang Z , Yin J , *et al* Pituitary adenylate cyclase‐activating polypeptide protects against β‐amyloid toxicity. Neurobiol Aging 2014; 35 **:** 2064–2071. 24726470 10.1016/j.neurobiolaging.2014.03.022

[74] Nakamachi T , Ohtaki H , Seki T , *et al* PACAP suppresses dry eye signs by stimulating tear secretion. Nat Commun 2016; 7 **:** 12034. 10.1038/ncomms12034 PMC4931240 27345595

[75] Takeda T , Matsushita T , Kurozumi M , *et al* Pathobiology of the senescence‐accelerated mouse (SAM). Exp Gerontol 1997; 32 **:** 117–127. 9088909 10.1016/s0531-5565(96)00068-x

[76] Vaudry D , Hamelink C , Damadzic R , *et al* Endogenous PACAP acts as a stress response peptide to protect cerebellar neurons from ethanol or oxidative insult. Peptides 2005; 26 **:** 2518–2524. 16009465 10.1016/j.peptides.2005.05.015 PMC4183202

[77] Szabadfi K , Atlasz T , Kiss P , *et al* Mice deficient in pituitary adenylate cyclase activating polypeptide (PACAP) are more susceptible to retinal ischemic injury *in vivo* . Neurotox Res 2012; 21 **:** 41–48. 21717232 10.1007/s12640-011-9254-y

[78] Sukhanova A , Poly S , Shemetov A , *et al* Implications of protein structure instability: from physiological to pathological secondary structure. Biopolymers 2012; 97 **:** 577–588. 22605549 10.1002/bip.22055

[79] Teng MH , Yin JY , Vidal R , *et al* Amyloid and nonfibrillar deposits in mice transgenic for wild‐type human transthyretin: a possible model for senile systemic amyloidosis. Lab Invest 2001; 81 **:** 385–396. 11310831 10.1038/labinvest.3780246

[80] Kojro E , Postina R , Buro C , *et al* The neuropeptide PACAP promotes the alpha‐secretase pathway for processing the Alzheimer amyloid precursor protein. FASEB J 2006; 20 **:** 512–514. 16401644 10.1096/fj.05-4812fje

[81] Postina R . Activation of α‐secretase cleavage. J Neurochem 2012; 120(suppl 1): 46–54. 10.1111/j.1471-4159.2011.07459.x 21883223

[82] Onoue S , Endo K , Ohshima K , *et al* The neuropeptide PACAP attenuates beta‐amyloid (1‐42)‐induced toxicity in PC12 cells. Peptides 2002; 23 **:** 1471–1478. 12182949 10.1016/s0196-9781(02)00085-2

[83] Chang YT , Wong CK , Chow KC , *et al* Apoptosis in primary cutaneous amyloidosis. Br J Dermatol 1999; 140 **:** 210–215. 10733268 10.1111/j.1365-2133.1999.02651.x

[84] Potter KJ , Scrocchi LA , Warnock GL , *et al* Amyloid inhibitors enhance survival of cultured human islets. Biochim Biophys Acta 2009; 1790 **:** 566–574. 19264107 10.1016/j.bbagen.2009.02.013

[85] Neree AT , Nguyen PT , Bourgault S . Cell‐penetrating ability of peptide hormones: key role of glycosaminoglycans clustering. Int J Mol Sci 2015; 16 **:** 27391–27400. 26580613 10.3390/ijms161126025 PMC4661883

[86] Chatenet D , Fournier A , Bourgault S . PACAP‐derived carriers: mechanisms and applications In Pituitary Adenylate Cyclase Activating Polypeptide – PACAP. Current Topics in Neurotoxicity, Reglodi D, Tamas A (eds), Vol 11 Springer Nature: New York, 2016; Ch 9, 133–148.

[87] Nishitsuji K , Uchimura K . Sulfated glycosaminoglycans in protein aggregation diseases. Glycoconj J 2017; 34 **:** 453–466. 28401373 10.1007/s10719-017-9769-4

[88] Marin‐Argany M , Lin Y , Misra P , *et al* Cell damage in light chain amyloidosis: fibril internalization, toxicity and cell‐mediated seeding. J Biol Chem 2016; 291 **:** 19813–19825. 27462073 10.1074/jbc.M116.736736 PMC5025671

