# Supplementary material for: Accelerated pre‐senile systemic amyloidosis in PACAP knockout mice – a protective role of PACAP in age‐related degenerative processes
Source: J Pathol. 2018 Jul 4;245(4):478–90. doi: 10.1002/path.5100 (PMC6055756; doi:10.1002/path.5100)
Supplement: Supplementary file 1 — Supplementary materials and methods [file PATH-245-478-s002.docx]

**Supplementary materials and methods**

Reference numbers refer to the main text reference list

***Histological analysis***

The following tissues/organs were removed and processed for histological evaluation from each animal: from the gastrointestinal system, the esophagus, stomach, small and large intestines, liver, pancreas, gallbladder, and salivary glands; from the endocrine glands, the thyroid, parathyroid, and adrenal and pituitary glands; from the respiratory system, the trachea, larynx, and lung; from the urogenital system, the kidney, urinary bladder, male genitals (testis, epididymis, seminal vesicle), and female genitals (ovary, uterus, vagina); from the musculoskeletal system, bones (femur, tibia), vertebral column, ribs, and skeletal muscle; from the nervous and sensory system, the brain, spinal cord, peripheral nerves (sciatic nerve), and eyeball were investigated. From the cardiovascular system, the heart and aorta were removed, but smaller vessels were also examined in other tissue samples. Lymphatic organs, such as spleen, thymus, and lymph nodes (mainly para-aortic), were also removed, and skin of the abdomen (including hypodermal adipose tissue), tail, and external ear were also included in the sample collection.

Besides amyloid deposits, no other pathological signs in the peripheral organs could be detected by light microscopic analysis (such as severe inflammation, cell necrosis or degeneration). The only exception was the liver, where amyloid-associated cellular degeneration could occasionally be observed.

***LMS-based microproteomic analysis***

Five-micrometer-thick sections of the FFPE block were deposited on polyethylene naphthalate LMD membrane slides (Leica Microsystems, Wetzlar, Germany), dried, and processed for deparaffination. Tissue sections were immersed in xylene, then three successive baths of 100% ethanol (Biosolve, Dieuze, France) and dried before LMD collection using a Leica LMD7000 with laser settings as described previously [1]. LMD was operated in bright field and it was possible to localize amyloid regions by correlation with Congo red-positive areas. Regions containing more than 95% of amyloid deposits were microdissected from the entire section. Amyloid regions were collected in a 0.6 ml Axygen tube cap (Fisher Scientific, Aalst, Belgium) and centrifuged at 16 000 relative centrifugal force. Twenty microliters of 10 mm pH 6 citric acid was added and heated at 99°C for 60 min for antigen retrieval, while shaking at 800 rpm in a Thermomixer (Eppendorf, Hamburg, Germany). Reduction/alkylation/reduction was followed by two steps of digestion with high trypsin concentrations. A first step consisted of digestion using 60 µg/ml trypsin in 50 mm NH_4_HCO_3_ overnight. Then 30 µg/ml trypsin was added together with acetonitrile (ACN) to adjust the solution to 80% ACN. Then a second step of digestion took place for 3 h in a more apolar environment. In this workflow, high concentrations of trypsin permit on-tissue digestion that compensates for the lack of prior protein extraction [37].

Since amyloid regions were collected from a whole tissue section and processed with a method designed initially for very small FFPE tissue pieces, only 1/10 of the volume of the proteolytic digest was used for further preparation and analysis. The sample was dried using a SpeedVac centrifuge (Savant^TM^ SPD 121P; Thermo Fisher Scientific, Waltham, MA, USA) and desalted using C_18_ Zip Tip cartridges (Milipore, Overijse, Belgium). The sample was resuspended in 10 µl of 100 mm NH_4_CHO_2_ buffer adjusted to pH 10 containing MPDS Mix1 (Waters, Zellik, Belgium) with 50 fmol of ADH protein digest. Peptides were separated by reverse-phase liquid chromatography using ultra performance liquid chromatography 2D nanoAcquity (Waters Corp, Milford, MA, USA) in two dimensions. Parameters for liquid chromatography–tandem mass spectrometry (LC–MS/MS) have been described previously [37].

The 2D-nanoUPLC system was configured for a reversed phase pH 10 followed by a reversed phase pH 3-based separation. The first dimension separation was made on an X-Bridge BEH C18 5 µm column (300 µm × 50 mm). For the second dimension, a trap column Symmetry C18 5 µm (180 µm × 20 mm) and an analytical column HSST3 C18 1.8 µm (75 µm × 250 mm) (Waters Corp) were used after an online dilution to lower pH values. The sample was loaded at 2 µl/min (20 mm ammonium formate solution adjusted to pH 10) on the first column and subjected to three isocratic elution steps (13.3%, 19%, and 65% ACN). Each eluted fraction was desalted on the trap column after a ten-times online dilution to pH 3 and subsequently separated on the analytical column: with a flow rate of 250 nl/min, solvent A (0.1% formic acid in water) and solvent B (0.1% formic acid in acetonitrile) with a linear gradient as follows: 0 min, 99% A; 5 min, 93% A; 140 min, 65% A. The following 40 min was used for cleaning and re-equilibration steps. The total run time for the second dimension separation of each of the fractions obtained by isocratic elution was 180 min.

The LC eluent was directly electrosprayed from the analytical column at 2.2 kV through the nanospray source. The chromatography system was coupled to a Thermo Scientific Q Exactive Hybrid Quadrupole-Orbitrap mass spectrometer (Thermo Fisher Scientific), programmed for data-dependent acquisition mode. A TopN-MS/MS method was used where *N* was set to 10. The parameters for MS spectrum acquisition were mass range from *m/z* 400 to 1750, resolution of 70 000 (full width at half maximum), automatic gain control (AGC) target of 1e10^6^ or maximum injection time of 200 ms. The parameters for MS/MS spectrum acquisition were isolation window of *m/z* 2.0, ion trap higher energy collision dissociation (HCD) fragmentations normalized collision energy of 25, resolution of 17 500, automatic gain control AGC target of 1e10^5^ or maximum injection time of 200 ms, dynamic exclusion: 10 s.

***Data processing***

MaxQuant version 1.5.2.8 was used for raw file analysis. MS/MS spectra were analyzed using the Andromeda search engine and the following settings: Uniprot mouse database release 2017-02 for interrogation, N-ter acetylation, oxidation of methionines, and deamidation (NQ) set as variable modifications, carbamidomethylation of the cysteines as fixed modification. The maximum number of missed cleavages was set at two and the minimal peptide length for identification was set at seven amino acids. At least two peptides per protein were required for identification. The main search tolerance was set at 4.5 ppm. Peptide spectrum match and protein false discovery rates were both set at 0.01. The table containing the protein identification and their intensity (‘proteinGroups’ file) was used to analyze the results. The identified amyloidosis-related proteins and their intensities were reported using GraphPad Prism 7 (GraphPad Software, La Jolla, CA, USA).

***MALDI mass spectrometry imaging (MSI)***

Deparaffinization in two changes of xylene was followed by rehydration in isopropanol and graded ethanol (Fisher Scientific, Schwerte, Germany). Antigen retrieval was performed using a decloaking chamber (Biocare Medical, Pacheco, CA, USA) in milliQ water. 0.05 µg/µl trypsin (Promega, Mannheim, Germany) solution in 20 mm ammonium bicarbonate (Sigma-Aldrich, Taufkirchen, Germany) was deposited onto tissues in eight layers using an automatic spraying device (TM Sprayer, HTX Technologies). Tissues were first subjected to protein digestion for 2 h in a humidity chamber and then sprayed with alpha-cyano-4-hydroxycinnamic acid (Sigma-Aldrich) matrix solution (10 mg/ml in 70:30 acetonitrile/water 1% TFA) in four layers.

***PCR analysis***

Samples were mechanically homogenized in Trizol (Applied Biosystems, Foster City, CA, USA) with a tissue grinder and were kept in room temperature for 1 h. After adding 20% RNase-free chloroform, samples were centrifuged at 4°C at 10 000 × *g* for 15 min. Samples were incubated in 500 µl of RNase-free isopropanol at −20°C for 1 h, then total RNA was harvested in RNase-free water and stored at −20°C. The assay mixture for reverse transcription contained 2 µg of RNA, 0.112 µm oligo(dT), 0.5 mm dNTP, 2.8 µm random primers, and 200 units of High Capacity RT (Applied Bio-Systems) in 1×RT buffer. Amplifications were performed in a thermal cycler (Labnet MultiGene™ 96-well Gradient Thermal Cycler; Labnet International, Edison, NJ, USA) in a final volume of 11 μl [containing 1 μl of forward and reverse primers (0.4 μm), 0.5 μl of dNTP (200 μm), and 5 units of Promega GoTaq^®^ DNA polymerase in 1× reaction buffer] as follows: 95°C, 2 min, followed by 35 cycles (denaturation, 94°C, 1 min; annealing at optimised temperatures as given in the supplementary material, Table S1 for 1 min; extension, 72°C, 90 s) and then 72°C, 10 min. The primer for *Apoa2* was designed according to ref 11. PCR products were analyzed by electrophoresis in 1.2% agarose gel containing ethidium bromide. Signals were acquired using a gel documenting system (FluorChem E, ProteinSimple, San José, CA, USA) using constant settings. *Gapdh* was used as internal control. Optical densities of PCR product signals were determined using ImageJ 1.40g freeware. All data are representative of at least three independent experiments. Statistical analysis was performed by unpaired Student’s *t*-test, where *p* < 0.05 was considered significant.

***Cytokine array analysis***

Kidney samples were homogenized in phosphate-buffered saline with protease inhibitors, then Triton X-100 was added to a final concentration of 1%. The array nitrocellulose membranes were blocked and sample homogenates containing 500 µg of protein were incubated overnight with reconstituted detection antibody cocktail. After washing and addition of streptavidin–horseradish peroxidase, plates were covered with a chemiluminescence reagent (Amersham Biosciences GE Healthcare, Budapest, Hungary) and sheets of film exposed. Developed films were then scanned and the mean intensities of the cytokine dot blots were calculated using ImageJ software.
