# Supplementary material for: Accelerated pre‐senile systemic amyloidosis in PACAP knockout mice – a protective role of PACAP in age‐related degenerative processes
Source: J Pathol. 2018 Jul 4;245(4):478–90. doi: 10.1002/path.5100 (PMC6055756; doi:10.1002/path.5100)
Supplement: Supplementary file 2 — Figure S1. Correlation between Congo‐red and the MALDI image of m/z 1228.7 from the mouse intestine tissue section presented in Figure 4. MALDI IMS distribution the ion peptide identified as Apoe with m/z value 1228.7 in a section of intestine with amyloidosis (right). Same section stained with Congo red after MALDI IMS (left); this Congo red panel is the same as shown in Figure 4. Figure S2. Representative data of three independent RT‐PCR reactions of amyloid specific protein mRNAs identified with mass spectrometry of aging WT and KO mice. Optical density of signals was measured and results were normalized to the optical density of controls. Gapdh was used as internal control. Numbers below the bands represent relative integrated densities of signals. The mRNA expression of Gsn, Lyz1, Apcs and Saa1 was augmented in the KO mice. However, that of B2m, Ighg1 and Igκ cC did not alter compared with WT animals. The mRNA expression of Ttr increased the most prominently and appeared with a three times stronger lane than in WT samples. Opposite to the protein level, expression of Apoa1 and Apoa4 mRNA was decreased in PACAP KO mice, while that of Apoe was increased. However, mRNA expression of Apoa2, the accumulation of which has been detected in Alzheimer disease, could not be shown. *p<0.05 compared to respective controls. Figure S3. Main laboratory serum parameters in young and old WT and PACAP KO mice. Values are given as mean±SEM, where *p<0.05 versus young mice. [file PATH-245-478-s001.docx]

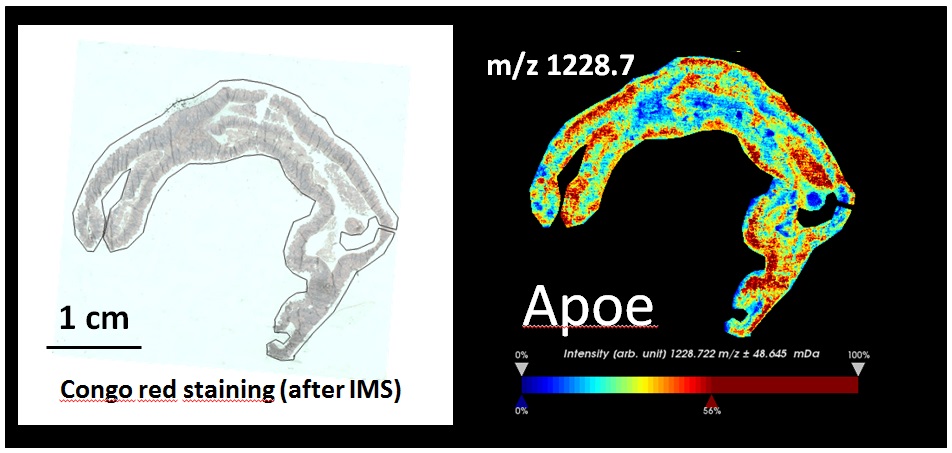


**Supplementary Figure S1. Correlation between Congo-red and the MALDI image of m/z 1228.7 from the mouse intestine tissue section presented in Figure 4.**

MALDI IMS distribution the ion peptide identified as Apoe with m/z value 1228.7 in a section of intestine with amyloidosis (right). Same section stained with Congo red after MALDI IMS (left); this Congo red panel is the same as shown in Figure 4.


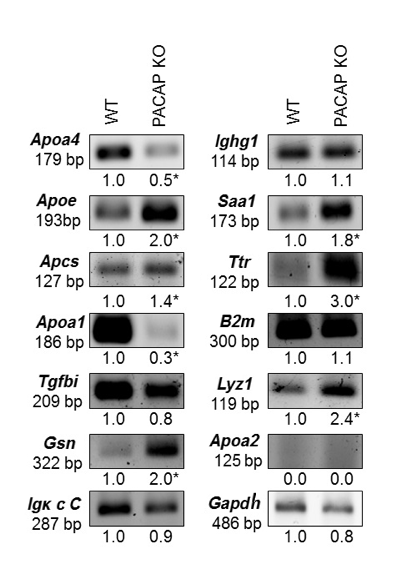


**Supplementary Figure S2. Representative data of three independent RT-PCR reactions of amyloid specific protein mRNAs identified with mass spectrometry of aging WT and KO mice.** Optical density of signals was measured and results were normalized to the optical density of controls. *Gapdh* was used as internal control. Numbers below the bands represent relative integrated densities of signals. The mRNA expression of *Gsn*, *Lyz1*, *Apcs* and *Saa1* was augmented in the KO mice. However, that of *B2m*, *Ighg1* and *Igκ cC* did not alter compared with WT animals. The mRNA expression of *Ttr* increased the most prominently and appeared with a three times stronger lane than in WT samples. Opposite to the protein level, expression of *Apoa1* and *Apoa4* mRNA was decreased in PACAP KO mice, while that of *Apoe* was increased. However, mRNA expression of *Apoa2*, the accumulation of which has been detected in Alzheimer disease, could not be shown. *p<0.05 compared to respective controls.


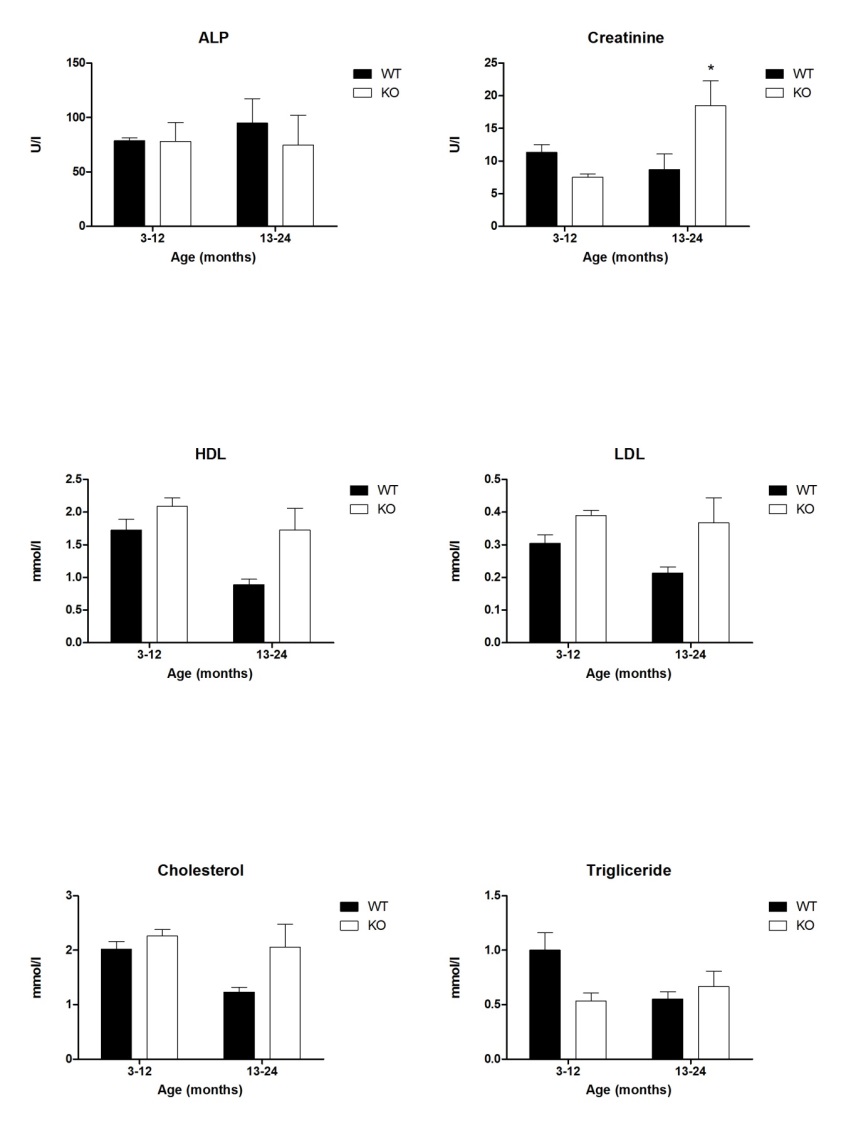


**Supplementary Figure S3. Main laboratory serum parameters in young and old WT and PACAP KO mice.** Values are given as mean±SEM, where *p<0.05 versus young mice.
