# Supplementary material for: Accelerated pre‐senile systemic amyloidosis in PACAP knockout mice – a protective role of PACAP in age‐related degenerative processes
Source: J Pathol. 2018 Jul 4;245(4):478–90. doi: 10.1002/path.5100 (PMC6055756; doi:10.1002/path.5100)
Supplement: Supplementary file 3 — Table S1. Nucleotide sequences, amplification sites, GenBank accession numbers, amplimer sizes and PCR reaction conditions for each mouse primer pair are shown [file PATH-245-478-s003.docx]

**Table S1.** Nucleotide sequences, amplification sites, GenBank accession numbers, amplimer sizes and PCR reaction conditions for each mouse primer pair are shown

| **Gene** | **Primer** | **Nucleotide sequence (5'→3')** | **GenBank ID** | **Annealing temperature** | **Amplimer size (bp)** |
| --- | --- | --- | --- | --- | --- |
| *Apoa1* | Sense | GTG AAG GAT TTC GCT AAT GTG (226–246) | [NM_009692.4](https://www.ncbi.nlm.nih.gov/nuccore/NM_009692.4) | 52°C | 186 |
|  | Antisense | CCA GAA GTC CCG AGT CAA T (393–411) |  |  |  |
| *Apoa4* | Sense | CCA ACA AAG TAA CCC AGA CG (660–679) | [NM_007468.2](https://www.ncbi.nlm.nih.gov/nuccore/NM_007468.2) | 56°C | 179 |
|  | Antisense | GTG CAG GTT GTC CAC ATT CTC  (818–838) |  |  |  |
| *Apoa2* | Sense | TGA AGC TTC TCG CAA TGG TCC CAC TGC TGG T  (98–128) | [NM_001305549.1](https://www.ncbi.nlm.nih.gov/nuccore/NM_001305549.1) | 59°C | 125 |
|  | Antisense | AGT CAT GCT CTG AAA GTA CTG TGT G  (223–248) |  |  |  |
| *Apoe* | Sense | GGG CAG TAC CGC AAC GA  (544–560) | [NM_001305819.1](https://www.ncbi.nlm.nih.gov/nuccore/NM_001305819.1) | 57°C | 193 |
|  | Antisense | GCT CAC GGA TGG CAC TCA  (719–736) |  |  |  |
| *Apcs* | Sense | CCT CTG GCA TTG TTG AA  (529–545) | [NM_011318.2](https://www.ncbi.nlm.nih.gov/nuccore/NM_011318.2) | 50°C | 127 |
|  | Antisense | CCT CCT CCG TAG TTA TCC  (559–575) |  |  |  |
| *B2m* | Sense | AAG ACC GTC TAC TGG GAT C  (382–400) | [NM_009735.3](https://www.ncbi.nlm.nih.gov/nuccore/NM_009735.3) | 52°C | 300 |
|  | Antisense | GAA GTA CAG AGG GTT TGG  (664–681) |  |  |  |
| *Gsn* | Sense | CTT TCG ATG CTG CTA CGC  (1317–1334) | [NM_001206367.1](https://www.ncbi.nlm.nih.gov/nuccore/NM_001206367.1) | 56°C | 322 |
|  | Antisense | TGG ACC ACT CGG CTC TG  (1622–1638) |  |  |  |
| *Ighg1* | Sense | AGA TGG CTC TTA CTT CGT C  (324–342) | V00795.1 | 51°C | 114 |
|  | Antisense | TCA GTA TGG TGG TTG TGC  (420–437) |  |  |  |
| *Igκ c C* | Sense | GGA GCC CTT CCT TGT TA  (162–178) | AJ880391.1 | 51°C | 287 |
|  | Antisense | TCA TAC TCG TCC TTG GTC  (431–448) |  |  |  |
| *Lyz1* | Sense | GCT ACC GTG GTG TCA AG  (140–156) | [NM_013590.4](https://www.ncbi.nlm.nih.gov/nuccore/NM_013590.4) | 49°C | 119 |
|  | Antisense | CTG AAA TAT CCC ATA GTC G  (240–258) |  |  |  |
| *Saa1* | Sense | TAT GAT GCT GCT CAA AGG  (211–228) | [NM_009117.3](https://www.ncbi.nlm.nih.gov/nuccore/NM_009117.3) | 50°C | 173 |
|  | Antisense | CCA GGA GGT CTG TAG TAA TT  (364–383) |  |  |  |
| *Tgfbi* | Sense | GAT GAA ATC CTG GTT AGC G  (1767–1785) | [NM_009369.4](https://www.ncbi.nlm.nih.gov/nuccore/NM_009369.4) | 52°C | 209 |
|  | Antisense | TCT CCT CGT TCT TGT GGT C  (1957–1975) |  |  |  |
| *Ttr* | Sense | TGG AAG ACA CTT GGC ATT T  (484–502) | [NM_013697.5](https://www.ncbi.nlm.nih.gov/nuccore/NM_013697.5) | 54°C | 122 |
|  | Antisense | GTG GTG CTG TAG GAG TAT GG  (586–605) |  |  |  |
| *Gapdh* | Sense | TGG CAA AGT GGA GAT TGT TG (69–88) | NM_008084 | 59°C | 486 |
|  | Antisense | GTC TTC TGG GTG GCA GTG AT (535–554) |  |  |  |
